# First Successful Targeted Mutagenesis Using CRISPR/Cas9 in Stably Transformed Grain Amaranth Tissue

**DOI:** 10.1111/pbi.70590

**Published:** 2026-02-11

**Authors:** Susanne K. Vollmer, Markus G. Stetter, Götz Hensel

**Affiliations:** ^1^ Centre for Plant Genome Engineering, Faculty of Mathematics and Natural Sciences Heinrich Heine University Düsseldorf Düsseldorf Germany; ^2^ Institute for Plant Sciences University of Cologne Cologne Germany; ^3^ Cluster of Excellence in Plant Sciences (CEPLAS) Heinrich Heine University Düsseldorf Düsseldorf Germany

**Keywords:** Amaranth, Betalain, callus, Caryophyllales, CRISPR/Cas, mutagenesis, orphan crop

## Abstract

Grain amaranth is a nutritionally rich, stress‐tolerant C_4_ dicot with considerable potential for climate‐resilient agriculture; however, efficient and reproducible protocols for stable transformation, regeneration, and CRISPR/Cas9‐mediated editing have not yet been established. CRISPR/Cas‐based genome editing is a cornerstone technology for accelerating the development of climate‐resilient, high‐yielding crops. Its effective application depends on robust, stable transformation procedures and CRISPR/Cas systems optimised for the target species. The absence of such tools remains a critical constraint for the genetic improvement of many promising yet underexplored crops. In this study, we edited key genes of the betalain biosynthesis pathway in grain amaranth (*Amaranthus hypochondriacus* L.) using the CasCADE modular cloning system, thereby demonstrating the feasibility of targeted mutagenesis in an orphan crop. We observed successful edits in up to 49% of transformed calli, resulting in deletions or insertions in the target genes. Our CRISPR/Cas9‐mediated editing paves the way for targeted molecular research and breeding of grain amaranth.

Genome editing using CRISPR/Cas is a key technology for speeding up breeding for climate‐resilient, high‐yielding crops (Scheben et al. 2017). However, efficient targeted mutagenesis requires implementing stable transformation methods and establishing a CRISPR/Cas setup suitable for the species of interest (Shan et al. 2020). The availability of such methods is a significant bottleneck to advancing many promising, albeit under‐researched, crops. Testing and establishing vectors for efficient application of CRISPR/Cas in non‐model crops could boost research and breeding of new valuable crops (Ye and Fan 2021). We edited key pathway genes in the betalain biosynthesis pathway of grain amaranth, i.e., *A. hypochondriacus* L., to prove how targeted mutagenesis can be implemented in an orphan crop using the CasCADE modular cloning system (Hoffie 2022). Grain amaranth is a resilient C_4_ dicot orphan crop with excellent nutritional composition. These properties make amaranth a well‐suited candidate to be bred as climate‐resilient crop (Joshi et al. 2018). However, no efficient and reproducible protocol for successful application of CRISPR/Cas9 or stable transformation and regeneration, has been demonstrated in *A. hypochondriacus* (Castellanos‐Arévalo et al. 2020).

Amaranth produces red and yellow betalains, which are specialised metabolites in *Caryophyllales* species (Timoneda et al. 2019). Betalains have been employed as reporters in molecular biology using the RUBY cassette, which consists of the three enzymes required to produce red betalains from tyrosine (He et al. 2020). Conversely, the pathway is well‐suited to evaluate the knockout efficiency through targeted mutagenesis in *A. hypochondriacus* , where the pigments naturally occur. Key enzyme genes in the betalain pathway are *AhCYP76AD2* and *AhCYP76AD5*, which catalyse the initial steps of betalain production. Natural knockouts of *AhCYP76AD2* have been shown to lack red colour (Winkler et al. 2024), suggesting its suitability as a visual reporter.

The foundations of successful CRISPR/Cas‐mediated targeted mutagenesis are efficient guide RNAs and suitable components to express the Cas9 enzyme and the gRNAs (Shan et al. 2020). The *Cas9* sequence should be adapted to the codon usage of the target species. The promoter that drives the expression of *Cas9* and the guides need to be highly active in the target species. Finally, the selection cassette has to function efficiently in the target species. To address these requirements in amaranth, we constructed a binary vector containing *Cas9* and a four‐guide cassette, using the CasCADE modular cloning system (Figure 1a,b, Figure S1, Table S1 and Appendix S1; Hoffie 2022). Amaranth plants successfully mutated in *AhCYP76AD2* should be deficient in betalains, facilitating the later detection of successfully edited regenerates. An additional gRNA not targeting the amaranth genome, but the *CYP76AD1* gene in the RUBY reporter cassette (He et al. 2020; gRNA 3) was included as a control (Figure 1).

**FIGURE 1:**
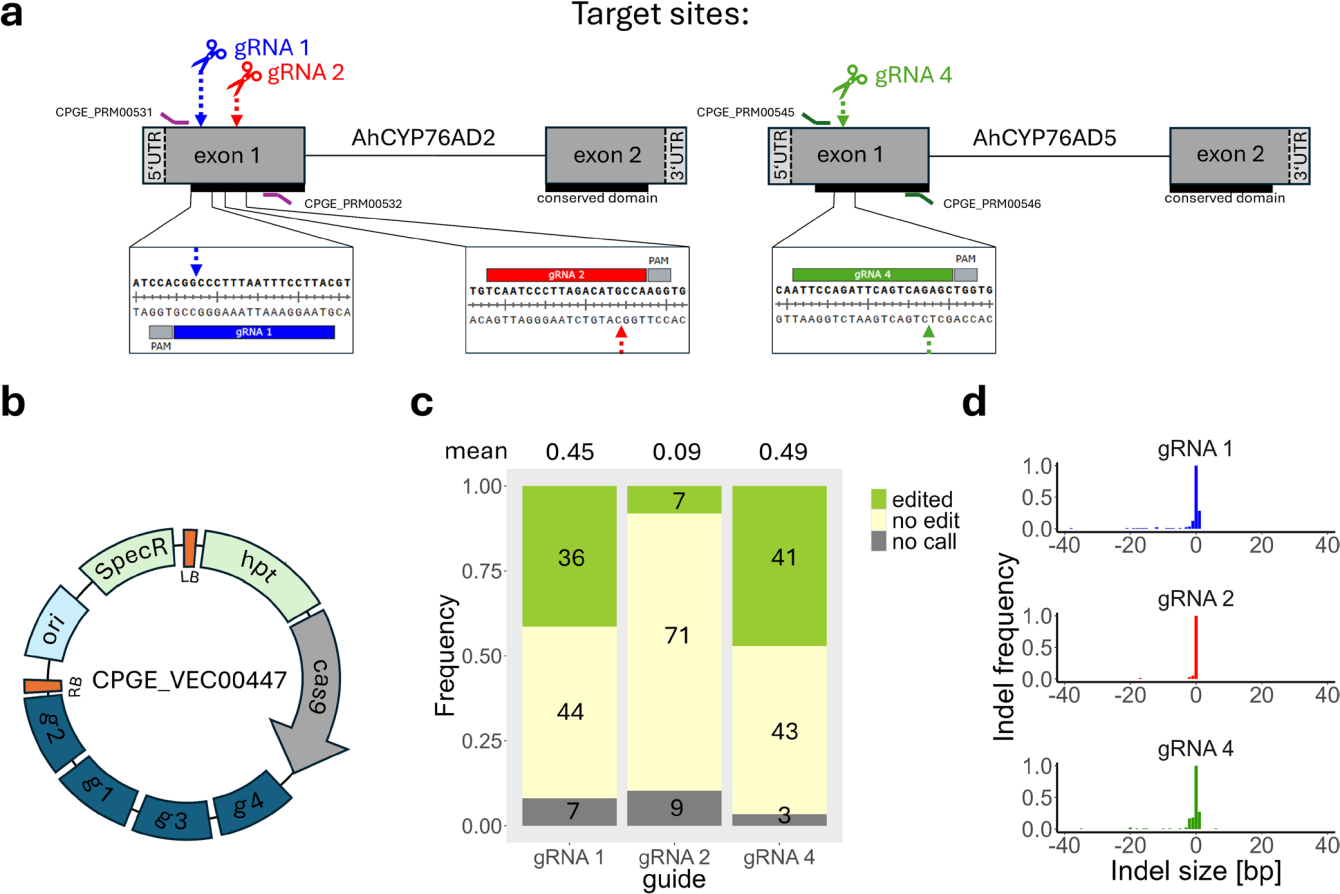
Overview of CRISPR/Cas9‐mediated targeted mutagenesis in grain amaranth. (a) Schematic structure of the target genes with the respective target sites of the guides in amaranth genes and the primers used for analysis. (b) Vector with four guides, two targeting the first exon of *AhCYP76AD2* (gRNA 1 and gRNA 2), one the first exon of *AhCYP76AD5* (gRNA 4) and one the *RUBY* reporter (gRNA 3). (c) Total number of mutations for all three guides. (d) Length and frequency of the observed InDels found in 74 lines.

The *Cas9* gene was codon‐optimised for the dicot *Arabidopsis thaliana* and driven by the parsley *Ubi4‐2* promoter (Hoffie 2022). Each gRNA was expressed separately by the *AtU6‐26* promoter (Hoffie 2022). For the transgenic tissue selection, the T‐DNA contains an intronized *hpt* gene driven by a *CaMV* doubled‐enhanced *35S* promoter to confer hygromycin resistance (Hoffie 2022). We cloned the CRISPR/Cas9 cassette into a binary vector via SfiI to enable *Rhizobium*‐mediated transformation (Hoffie 2022; Figure 1b).

Next, we established callus transformation, which enables the highly efficient production of stable transgenic tissue in multiple‐grain amaranth species. The results were obtained from two independent transformation replicates, batch 1 and batch 2. A random subset of 23 calli from batch 1 and all from batch 2, treated with *R. radiobacter* carrying the CRISPR/Cas9 vector, were analysed for edits. Briefly, 5‐week‐old calli of *A. hypochondriacus* were transformed with the CRISPR/Cas9 vector using *R. radiobacter* strain GV3101. For co‐culture, calli were incubated on filter paper for 3 days before being transferred to a callus induction medium with selection. All calli treated with *Rhizobium* carrying the CRISPR/Cas9 vector (42/42 for batch 1 and 65/65 for batch 2) grew new resistant calli on the selection medium.

In contrast, only two (1/21 for batch 1 and 1/32 for batch 2) of the non‐treated calli survived the selection and propagated new callus, indicating the high success of transformation. After multiple rounds of selection, callus material was sampled for genotyping and analysis (Figure S2). Genomic DNA (gDNA) was extracted from 87 calli of the two independent batches.

We sampled individual calli; however, callus tissue can contain cells from independent transformation events with different edits. Therefore, each gDNA included a pool of wild‐type and edited alleles. To increase the sensitivity for detecting edited alleles using Sanger sequencing, we employed restriction enzyme digestion‐suppressed PCR (RE‐PCR) for two of the guides (gRNA 1 and gRNA 4) in the amaranth genome. Through the deconvolution of the obtained chromatograms, we found edits in 49% (gRNA 4) and 45% (gRNA 1) of the analysed calli (Figure 1c). In contrast, for gRNA 2, a lack of a restriction enzyme recognition site at the Cas9‐mediated cut site precluded RE‐PCR; edits could still be detected in 9% of the samples. Among all samples with data available for the three sites, 24.3% were edited for both target genes (*AhCYP76AD5 and AhCYP76AD2*) and 5.4% for all three guide RNA positions.

To ensure complete knockouts for downstream analysis of the target gene, targeted mutagenesis should achieve a diverse set of mutations, including larger insertions and deletions (InDels). According to the deconvolution data, most edits resulted in the deletion or insertion of one base pair (Figure 1d). However, for each guide, edits with larger deletions were also found (max. 35 bp for gRNA 4, 17 bp for gRNA 2, 38 bp for gRNA 1; Figure S3). To confirm the results from the deconvolution, we cloned amplicons of the target region of six random calli from gRNA 4 and five calli from gRNA 1 into pGEM‐Teasy (Promega) and sequenced eight colonies from each. In the majority of cases, the frequency for a particular type of InDels was higher in the subcloned colonies compared to the predictions (Table S2).

Our established callus transformation protocol enabled us to apply CRISPR/Cas9 in stable transformed tissue. Together with regeneration methods developed for *Amaranthus* (Castellanos‐Arévalo et al. 2020), and callus‐free methods, our transformation protocol can accelerate research and improvement of the crop (Maher et al. 2020). The presented results show the first successful targeted mutagenesis in grain amaranth using CRISPR/Cas9. Using the CasCADE modular cloning system and our established callus transformation, we achieved an approximately 50% editing efficiency for each of the two target genes. This paves the way for genome editing in grain amaranth for research and breeding of this orphan crop. Moreover, it may also guide the design of CRISPR systems for other species of the *Caryophyllales*, where reports of successful edits are still scarce.

## Author Contributions

M.G.S. and G.H. supervised the project. S.K.V. performed the experiments and analysed the data. S.K.V., M.G.S. and G.H. discussed and interpreted the data. S.K.V. wrote the draft of the manuscript and prepared the figures. All authors edited and approved the manuscript.

## Conflicts of Interest

The authors declare no conflicts of interest.

## Supporting information


**Figure S1:** Plasmid map of the CRISPR/Cas9 vector used for the targeted mutagenesis (CPGE_VEC00447).
**Figure S2:** Genotyping of genomic DNA of 74 calli for the presence of the *Cas9* gene using primer pairs mentioned in Table S1. Samples with a visible amplification are marked with green (67/74).
**Figure S3:** Gel electrophoresis of target amplicons for *AhCYP76AD5* (gRNA 4) using primer pairs mentioned in Suppl Table 1. Genomic DNA was amplified from wild‐type (WT) and edited callus (PCR). To enrich for the edited alleles, the DNA of the edited callus was digested with Hpy188I before the PCR (RE‐PCR) to visualise better the 35 bp deletion at the target site of gRNA 4. MW—100 bp molecular weight ladder.
**Table S1:** Primers used for cloning and genotyping.
**Table S2:** Comparison of the obtained edit frequency from subcloning and deconvolution. Note that subcloning is a sign of higher quality; however, it is not suitable for precisely determining edit frequencies due to the limited number of samples. The frequency of mutations was calculated as the percentage of samples with edits among all sequenced samples, or refers to the inferred rate of these edits in the deconvoluted Sanger sequencing chromatograms.
**Appendix S1:** Annotated DNA sequence of the CRISPR/Cas9 vector (CPGE_VEC00447).

## Data Availability

The data that support the findings of this study are available within the article and its supplementary materials.
