# Supplementary material for: First Successful Targeted Mutagenesis Using CRISPR/Cas9 in Stably Transformed Grain Amaranth Tissue
Source: Plant Biotechnol J. 2026 Feb 11;24(6):3594–6. doi: 10.1111/pbi.70590 (PMC13205832; doi:10.1111/pbi.70590)
Supplement: Supplementary file 1 — Figure S1: Plasmid map of the CRISPR/Cas9 vector used for the targeted mutagenesis (CPGE_VEC00447). Figure S2: Genotyping of genomic DNA of 74 calli for the presence of the Cas9 gene using primer pairs mentioned in Table S1. Samples with a visible amplification are marked with green (67/74). Figure S3: Gel electrophoresis of target amplicons for AhCYP76AD5 (gRNA 4) using primer pairs mentioned in Suppl Table 1. Genomic DNA was amplified from wild‐type (WT) and edited callus (PCR). To enrich for the edited alleles, the DNA of the edited callus was digested with Hpy188I before the PCR (RE‐PCR) to visualise better the 35 bp deletion at the target site of gRNA 4. MW—100 bp molecular weight ladder. Table S1: Primers used for cloning and genotyping. Table S2: Comparison of the obtained edit frequency from subcloning and deconvolution. Note that subcloning is a sign of higher quality; however, it is not suitable for precisely determining edit frequencies due to the limited number of samples. The frequency of mutations was calculated as the percentage of samples with edits among all sequenced samples, or refers to the inferred rate of these edits in the deconvoluted Sanger sequencing chromatograms. Appendix S1: Annotated DNA sequence of the CRISPR/Cas9 vector (CPGE_VEC00447). [file PBI-24-3594-s001.pdf]

**Title:** First Successful Targeted Mutagenesis Using CRISPR/Cas9 in Stably Transformed Grain Amaranth Tissue

**Author information:**
Susanne K Vollmer[1,2,3], Markus G Stetter[2,3] and Götz Hensel[1,3*]
[*] corresponding author

**Affiliation:**
[1] Heinrich Heine University Düsseldorf, Faculty of Mathematics and Natural Sciences, Centre for Plant Genome Engineering, Düsseldorf, 40225, Germany
[2] Institute for Plant Sciences, University of Cologne, Cologne, 50674, Germany
[3] Cluster of Excellence in Plant Sciences (CEPLAS), Heinrich Heine University Düsseldorf, 40225, Germany

**Corresponding author:**
henselg@hhu.de

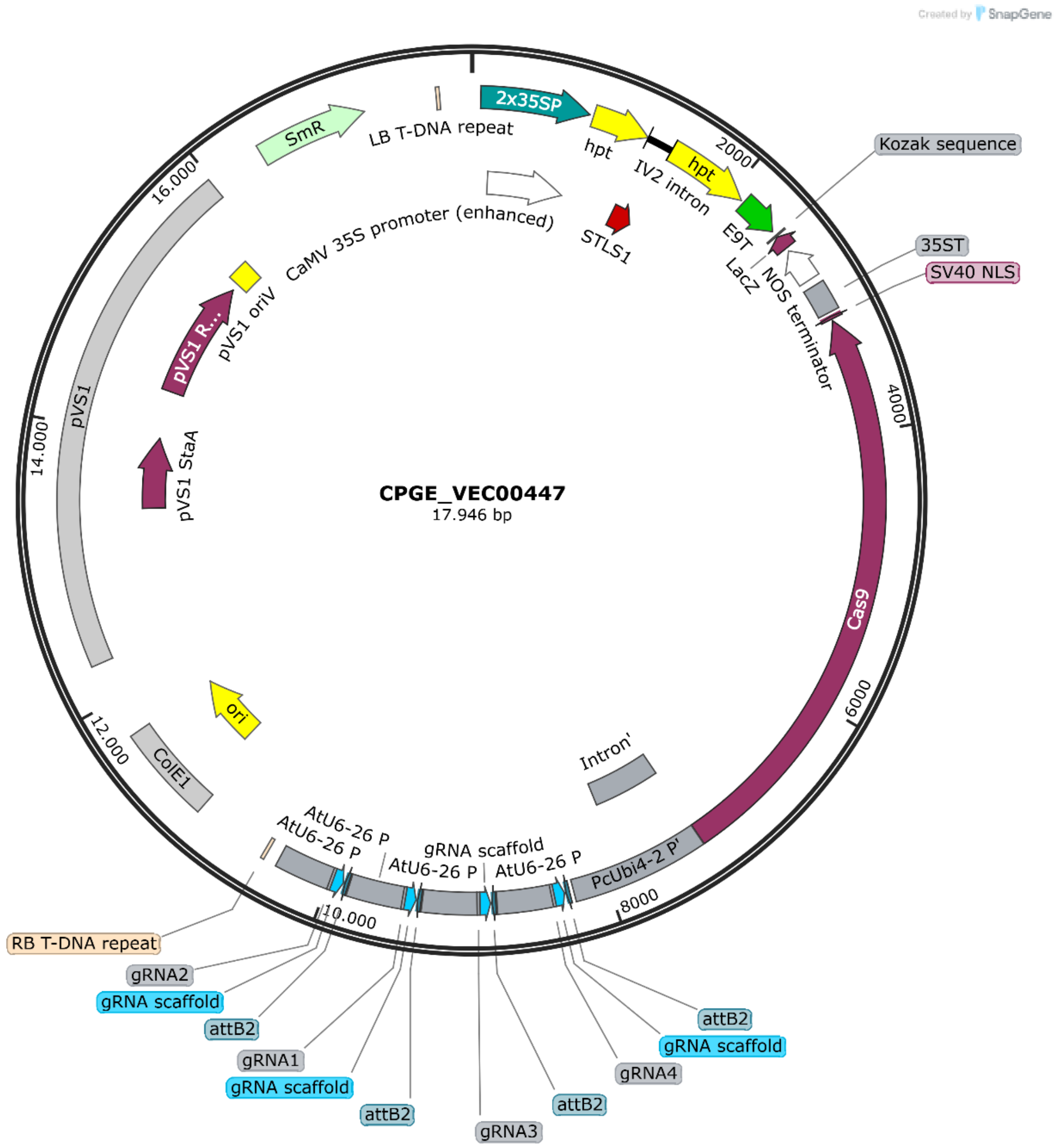


**Supplemental Figure 1:** Plasmid map of the CRISPR/Cas9 vector used for the targeted mutagenesis (CPGE_VEC00447).

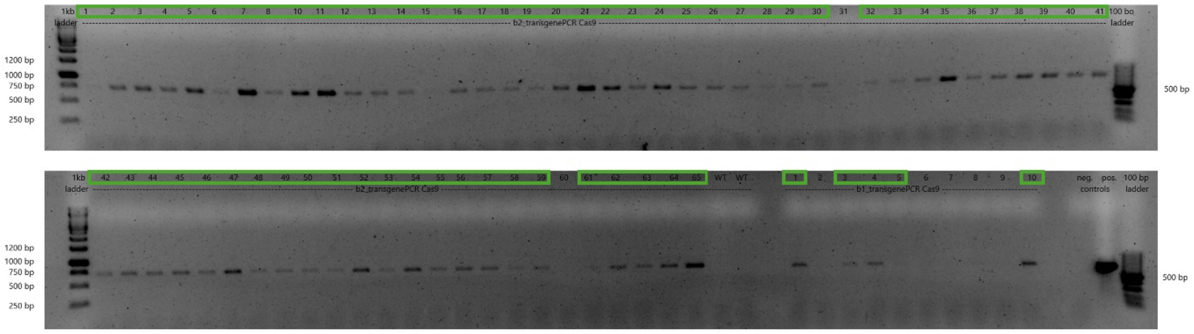


**Supplemental Figure 2:** Genotyping of genomic DNA of 74 calli for the presence of the Cas9 gene using primer pairs mentioned in Suppl Table 1. Samples with a visible amplification are marked with green (67/74).

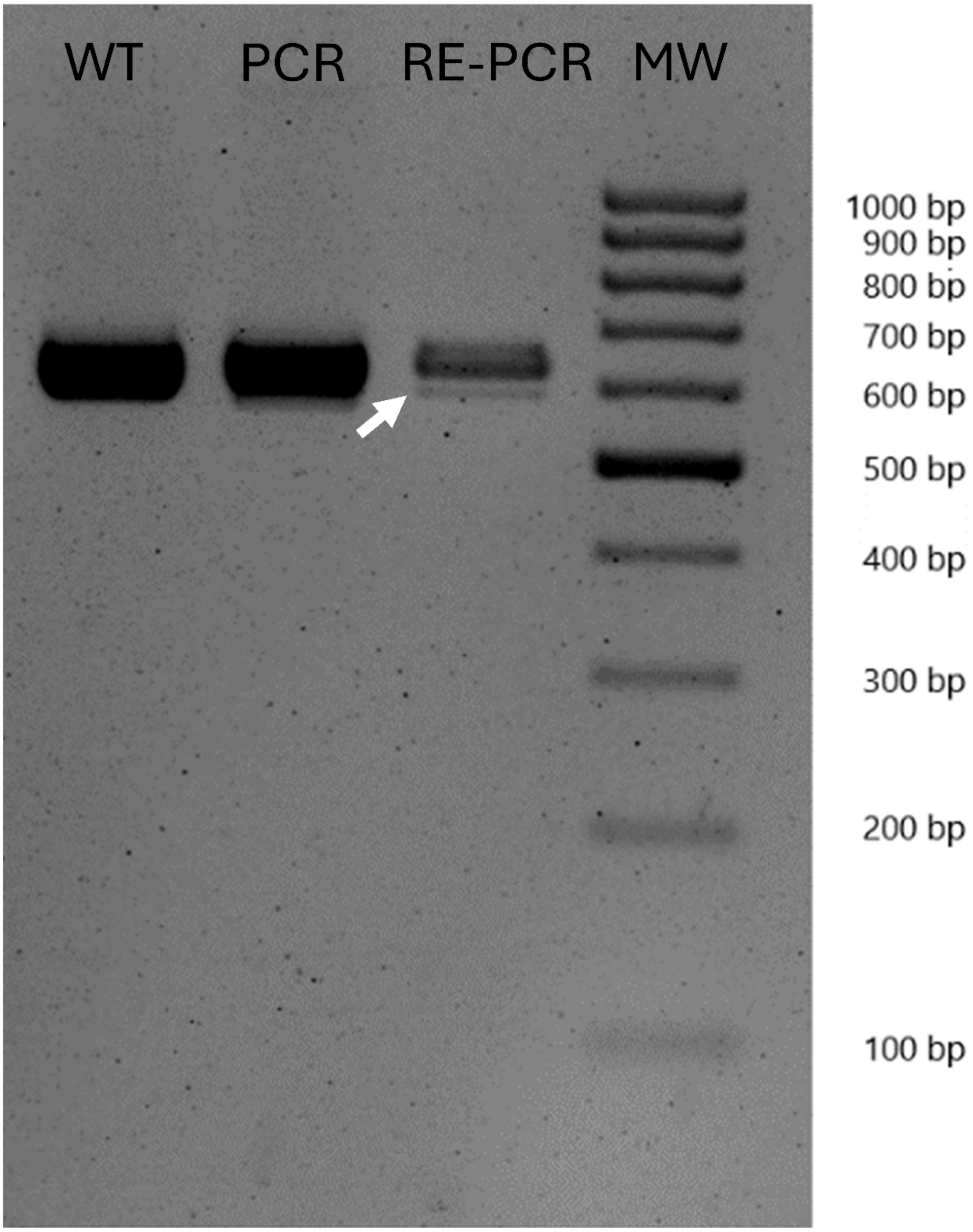


**Supplemental Figure 3:** Gel electrophoresis of target amplicons for AhCYP76AD5 (gRNA 4) using primer pairs mentioned in Suppl Table 1. Genomic DNA was amplified from wild-type (WT) and edited callus (PCR). To enrich for the edited alleles, the DNA of the edited callus was digested with Hpy188I before the PCR (RE-PCR) to visualize better the 35 bp deletion at the target site of gRNA 4. MW – 100 bp molecular weight ladder.

**Supplemental Table 1:** Primers used for cloning and genotyping.

| Primer | Sequence (5‘- 3‘) | Purpose |
|---|---|---|
| CPGE_PRM00523 | ATTGgcaatcccttagacatgcca | To obtain gRNA 2 sequence with overhangs |
| CPGE_PRM00524 | AAACtggcatgtctaagggattgc | |
| CPGE_PRM00529 | AAACcggccctttaatttccttat | To obtain gRNA 1 sequence with overhangs |
| CPGE_PRM00530 | ATTGataaggaaattaaagggccg | |
| CPGE_PRM00535 | AAACccgaccgtgttcttgctccc | To obtain gRNA 3 sequence with overhangs |
| CPGE_PRM00536 | ATTGgggagcaagaacacggtcgg | |
| CPGE_PRM00541 | ATTGattccagattcagtcagagc | To obtain gRNA 4 sequence with overhangs |
| CPGE_PRM00542 | AAACgctctgactgaatctggaat | |
| CPGE_PRM00531 | tcatggataatgcaaccctagc | Amplification of target region in *AhCYP76AD2* covering gRNA 1 and gRNA 2 |
| CPGE_PRM00532 | tgtttcaattcttgagattcatgaga | |
| CPGE_PRM00545 | tggtttcatctgtagaagtagcga | Amplification of target region in *AhCYP76AD5* covering gRNA 4 |
| CPGE_PRM00546 | cacaaggagatgattaatttcactca | |
| CPGE_PRM00037 | cagctcgtgcagacctacaac | Genotyping for the presence of the *Cas9* gene in the genome |
| CPGE_PRM00038 | tgccttctaaggatagcgtg | |

**Supplemental Table 2:** Comparison of the obtained edit frequency from subcloning and deconvolution. Note that subcloning is a sign of higher quality; however, it is not suitable for precisely determining edit frequencies due to the limited number of samples. The frequency of mutations was calculated as the percentage of samples with edits among all sequenced samples, or refers to the inferred rate of these edits in the deconvoluted Sanger sequencing chromatograms.

| **Target site_callus number** | **N subcloned analyzed by Sanger sequencing** | **Frequency and modification(s) detected in subcloned fragments** | **Results of chromatogram deconvolution using ICE** |
|---|---|---|---|
| gRNA1_5 | 8 | 25% 1 bp insertion | 19% 1 bp insertion |
| gRNA1_12 | 6 | 0% efficiency | 5% 1 bp insertion;<br>3% 18 bp deletion |
| gRNA1_24 | 6 | 0% efficiency | 4% 7 bp deletion;<br>1% 7 bp deletion |
| gRNA1_25 | 7 | 57% 38 bp deletion | 25% 38 bp deletion;<br>2% 1 bp insertion;<br>1% 6 bp deletion;<br>1% 5 bp deletion |
| gRNA1_64 | 5 | 20% 1 bp insertion | 19% 1 bp insertion;<br>8% 1 bp deletion |
| gRNA4_5 | 8 | 25% 1 bp insertion;<br>12.5% 35 bp deletion | 7% 1 bp insertion;<br>1% 2 bp deletion |

| | | | |
|---|---|---|---|
| gRNA4_12 | 8 | 37.5% 1 bp insertion | 9% 1 bp insertion |
| gRNA4_17 | 8 | 25% 1 bp deletion | 15% 1 bp insertion |
| gRNA4_25 | 8 | 25% 35 bp deletion | 8% 35 bp deletion;<br>7% 1 bp insertion |
| gRNA4_52 | 8 | 12.5% 1 bp deletion;<br>12.5% 2 bp deletion;<br>12.5% 1 bp insertion | 11% 1 bp deletion;<br>5% 1bp insertion;<br>1% 2 bp deletion;<br>1 % 16 bp deletion |
| gRNA4_64 | 6 | 0% efficiency | 0% efficiency |

**Supplemental Appendix 1:** Annotated DNA sequence of the CRISPR/Cas9 vector (CPGE_VEC00447)

Labelling: d35S promoter intronized hpt E9 terminator NOS terminator 35S terminator Cas9 PcUbi4-2 promoter, gRNA scaffold, AtU6-26 promoter, gRNA1, gRNA2, gRNA3, gRNA4, SpecR, ColE1 ori, pVS1 ori, **left and right border**

CGTGTCTACATTCACGTCCAAATGGGGGCTTAGATGAGAAACTTCACGATCGGCTCTAGATCGGGCCA
ACATGGTGGAGCACGACACTCTCGTCTACTCCAAGAATATCAAAGATACAGTCTCAGAAGACCAAAGG
GCTATTGAGACTTTTCAACAAAGGGTAATATCGGGAAACCTCCTCGGATTCCATTGCCCAGCTATCTG
TCACTTCATCAAAAGGACAGTAGAAAAGGAAGGTGGCACCTACAAATGCCATCATTGCGATAAAGGAA
AGGCTATCGTTCAAGATGCCTCTGCCGACAGTGGTCCCAAAGATGGACCCCCACCCACGAGGAGCATC
GTGGAAAAAGAAGACGTTCCAACCACGTCTTCAAAGCAAGTGGATTGATGTGATAACATGGTGGAGCA
CGACACTCTCGTCTACTCCAAGAATATCAAAGATACAGTCTCAGAAGACCAAAGGGCTATTGAGACTT
TTCAACAAAGGGTAATATCGGGAAACCTCCTCGGATTCCATTGCCCAGCTATCTGTCACTTCATCAAA
AGGACAGTAGAAAAGGAAGGTGGCACCTACAAATGCCATCATTGCGATAAAGGAAAGGCTATCGTTCA
AGATGCCTCTGCCGACAGTGGTCCCAAAGATGGACCCCCACCCACGAGGAGCATCGTGGAAAAAGAAG
ACGTTCCAACCACGTCTTCAAAGCAAGTGGATTGATGTGATATCTCCACTGACGTAAGGGATGACGCA
CAATCCCACTATCCTTCGCAAGACCTTCCTCTATATAAGGAAGTTCATTTCATTTGGAGAGGACACGC
TGAAATCACCAGTCTCTCTCTACAAATCTATCTCTCTCGAGGGCCCCGGGGGGCAATAAGATATGAAA
AAGCCTGAACTCACCGCGACGTCTGTCGAGAAGTTTCTGATCGAAAAGTTCGACAGCGTCTCCGACCT
GATGCAGCTCTCGGAGGGCGAAGAATCTCGTGCTTTCAGCTTCGATGTAGGAGGGCGTGGATATGTCC
TGCGGGTAAATAGCTGCGCCGATGGTTTCTACAAAGATCGTTATGTTTATCGGCACTTTGCATCGGCC
GCGCTCCCGATTCCGGAAGTGCTTGACATTGGGGAATTCAGCGAGAGCCTGACCTATTGCATCTCCCG
CCGTGCACAGGGTGTCACGTTGCAAGACCTGCCTGAAACCGAACTGCCCGCTGTTCTGCAGCCGGTCG
CGGAGGCCATGGATGCGATCGCTGCGGCCGATCTTAGCCAGACGAGCGGGTTCGGCCCATTCGGACCG
CAAGGTAAGTTTCTGCTTCTACCTTTGATATATATATAATAATTATCATTAATTAGTAGTAATATAAT
ATTTCAAATATTTTTTTCAAAATAAAAGAATGTAGTATATAGCAATTGCTTTTCTGTAGTTTATAAGT
GTGTATATTTTAATTTATAACTTTTCTAATATATGACCAAAATTTGTTGATGTGCAGGTATCGGTCAA
TACACTACATGGCGTGATTTCATATGCGCGATTGCTGATCCCCATGTGTATCACTGGCAAACTGTGAT
GGACGACACCGTCAGTGCGTCCGTCGCGCAGGCTCTCGATGAGCTGATGCTTTGGGCCGAGGACTGCC
CCGAAGTCCGGCACCTCGTGCACGCGGATTTCGGCTCCAACAATGTCCTGACGGACAATGGCCGCATA
ACAGCGGTCATTGACTGGAGCGAGGCGATGTTCGGGGATTCCCAATACGAGGTCGCCAACATCTTCTT
CTGGAGGCCGTGGTTGGCTTGTATGGAGCAGCAGACGCGCTACTTCGAGCGGAGGCATCCGGAGCTTG

CAGGATCGCCGCGGCTCCGGGCGTATATGCTCCGCATTGGTCTTGACCAACTCTATCAGAGCTTGGTT
GACGGCAATTTCGATGATGCAGCTTGGGCGCAGGGTCGATGCGACGCAATCGTCCGATCCGGAGCCGG
GACTGTCGGGCGTACACAAATCGCCCGCAGAAGCGCGGCCGTCTGGACCGATGGCTGTGTAGAAGTAC
TCGCCGATAGTGGAAACCGACGCCCCAGCACTCGTCCGAGGGCAAAGGAATAGAGTAGATGCCGACGC
GTTCGAGTATTATGGCATTGGGAAAACTGTTTTTCTTGTACCATTTGTTGTGCTTGTAATTTACTGTG
TTTTTTATTCGGTTTTCGCTATCGAACTGTGAAATGGAAATGGATGGAGAAGAGTTAATGAATGATAT
GGTCCTTTTGTTCATTCTCAAATTAATATTATTTGTTTTTTCTCTTATTTGTTGTGTGTTGAATTTGA
AATTATAAGAGATATGCAAACATTTTGTTTTGAGTAAAAATGTGTCAAATCGTGGCCTCTAATGACCG
AAGTTAATATGAGGAGTAAAACACTGAAGCCTGCAGGCATGCAAGCTGATCCACTAGAGGCCATGGcg
gccacggaattacgccaagcttgcatgcaggcctctgcagtcgacgggcccgggatccgatatctaga
tgcattcgcgaggtaccgagctcgaattcactggccgtttcgtctactgagcgtaaaaaccggtcccg
atctagtaacatagatgacaccgcgcgcgataatttatcctagtttgcgcgctatattttgttttcta
tcgcgtattaaatgtataattgcgggactctaatcataaaaacccatctcataaataacgtcatgcat
tacatgttaattattacatgcttaacgtaattcaacagaaattatatgataatcatcgcaagaccggc
aacaggattcaatcttaagaaactttattgccaaatgtttgaacgatcgagctcggggaaattcggat
ccccaatacttgtatggaggcctgagccgtacgggtcactggattttggttttaggaattagaaattt
tattgatagaagtattttacaaatacaaatacatactaagggtttcttatatgctcaacacatgagcg
aaaccctataagaaccctaattcccttatctgggaactactcacacattattctggagaaaaatagag
agagatagatttgtagagagagactggtgattttgcggactctagcggtcggcatctactgcggccg
cacctcaaaccttcctcttcttcttaggatcagcccttgaatcaccaccgagctgtgagagatcgatc
ctagtctcgtagagtccagtgatagactgatggatgagggtagcatcgagcacttctttggtagaggt
gtatctcttcctatcgatggttgtatcgaagtacttgaaagcagcaggagcaccgaggttggtaaggg
tgaagagatggatgatgttctctgcctgttccctgataggcttatctctgtgcttgttgtaagcagac
aacaccttatcgaggtttgcatcagcgaggatcaccctttagagaactcagagatctgctcgatgat
ctcatccaagtagtgcttgtgctgctcaacgaaaagttgcttctgctcgttatcttctggagatccct
tcaacttctcgtagtgagaagcgaggtaaagaaagttaacgtacttagatgggagagcaagctcgttt
ccctttgaagctcaccagcagaagcgagcatcctctttctaccgttctcgagttcgaagagtgagta
ctttgggagcttgatgatgagatccttcttaacctctttgtatcccttagcctcgaggaaatcgattg
ggttcttctcgaaagatgacctttccatgatagtgattccgagaagttccttaacagacttgagcttc
ttactctttcccttctcaaccttagccacaacgagaacagagtaagccacggtaggagaatcgaaacc
accgtatttcttagggtcccaatccttcttcctagcaatgagcttatcagagttcctcttagggagga
tagactctttagagaatccaccggtctgcacctcggtttcttaacgatgttcacctgtggcatagag
agcacctttctaacggtagcgaaatcccttcccttatcccacacgatctcacctgtttcaccgtttgt
ctcgatgagtggcctctttctgatctcaccgttagcgagggtaatctcggtcttgaagaaattcatga
tgttagagtagaagaaatacttagcggtagcctttccgatctcttgctcagacttagcgatcatcttc
ctcacatcgtacaccttgtaatcaccgtacacgaactctgactcgagcttaggatacttcttgatgag
agcggttccaacaacagcgttaaggtaagcatcgtgagcgtggtggtagttgttgatttccctcacct
tgtagaattggaaatcctttctgaaatcagacacgagctttgacttgagggtgataaccttcacttcc
ctgatcaacttatcgttctcatcgtacttggtgttcatcctagaatcgaggatctgtgcaacgtgctt
agtgatctgcctggtttccacaagctgcctcttgatgaatcctgccttatccaattcagagagtcctc
ccctctcagccttagtcaagttatcgaactttctctgagtgatgagcttagcgttgaggagctgcctc
caatagttcttcatttcttcacaacctcttcacttggcacgttatcactcttaccctgttcttatc
agacctggtgagcaccttgttatcgatagaatcatccttcaagaatgactgtggcacgatatgatcaa
catcgtaatcagagagcctgttgatatccaactcttgatccacatacatatcccttccgttctggagg
tagtagaggtagagcttctcattctggagctgagtgttctcaacagggtgctctttgaggatctgaga
tccaagctctttgataccttcctcgatcctcttcatcctttccctagagttcttctgtcccttctgag
tggtctggttctctctagccatttcgatcacgatgttctcaggcttatgccttcccatcaccttcacc
aactcatccacaaccttcacagtctggaggattcccttcttgattgcaggagatccagcgaggttagc
gatatgctcatggagactatcaccctgtcctgaaacctgagccttctggatatcctctttaaaggtga

gagaatcatcgtggatgagctgcatgaagtttctgttagcgaatccatcagacttgaggaaatcaagg
attgtctttccagactgcttatccctgattccgttaatgagctttcttgagagccttccccaaccagt
gtatcttcttctcttcaactgcttcatcaccttatcatcgaagagatgagcgtaggtcttgagccttt
cttcaatcatctctctatcttcaaagagggtgagggtaagaacgatatcctccaagatatcctcgttt
tcctcgttatccaagaaatccttatccttaatgatcttgaggagatcgtggtaggttccgagagatgc
gttgaacctatcctcaacaccagaaatctcaactgaatcgaagcactcgattttcttgaagtaatcct
ctttgagctgcttcacggtcacctttctgttggtcttgaacaagagatcaacgatagccttcttttgc
tcacctgacaaaaaagcaggcttcctcattccctcggtcacgtacttaaccttggtcaactcgttgta
cacggtgaagtactcgtagagcaaagagtgcttagggagcaccttctcgtttggaaggttcttatcga
agttggtcatcctctcgatgaaagactgagcactagcacccttatccaccacctcttcgaagttccaa
ggggtgatggtttcctcagactttctggtcatccaagcgaatcttgagtttcctctagcgagaggtcc
cacgtagtaagggattctgaaggtgagaatcttctcaatcttttccctgttatccttgaggaatgggt
agaaatcctcttgccttctaaggatagcgtgcaactctccgaggtggatctgatgagggatagatccg
ttatcgaaggtcctctgctttctgagaagatcctctctattgagcttcacgaggagttcctcggttcc
atccatcttctcgaggataggcttgatgaacttgtagaactcttcttgagatgcaccaccatcgatgt
aaccagcgtatccgttcttagactgatcgaagaaaatctctttgtacttctctgggagctgctgtcta
acaagagccttgagaagtgtgagatcctggtggtgctcatcgtatctcttgatcatagaagctgagag
tggagccttggtgatctcggtgttcactctgaggatatcactgaggaggatagcatcagagaggttct
tagcagcgaggaacaaatcagcgtactgatctccgatctgagcgaggaggttatcgagatcatcatcg
taggtatcctttgagagctggagctttgcatcctcagcgagatcgaagttagacttgaagttaggggt
gagtccgagagagagagagcgatcaagtttccgaaaagtccgttcttcttctcaccagggagctgagcaa
tgaggttctcaagccttcttgacttagagagcctagcagagaggatagccttagcatccacacctgaa
gcgttgatagggttctcttcgaaaagctggttgtaggtctgcacgagctggatgaacaacttatccac
atcagagttatcagggttgagatcaccctcgatgaggaagtgtcctctgaacttgatcatgtgagcga
gagcgaggtagatgagcctgagatcagccttatcagtagaatcaacgagcttctttctgaggtggtag
atagtagggtacttctcgtggtatgccacctcatcaacgatgtttccgaagatagggtgcctctcgtg
cttcttatcttcttccacgaggaatgactcttcgagcctgtggaagaatgaatcatccactttagcca
tctcgttagagaagatctcttggaggtagcagatcctgttctttcttctggtgtaccttcttctagcg
gttctcttgagtctggtagcctcagcagtttcaccagaatcgaagaggagagcaccgataaggttttt
cttgatagagtgcctatcggtgtttccgagaaccttgaacttcttagatggcaccttgtactcatcgg
tgatcacagcccatcccacagagttagttccgatatcgagtccgatagagtacttcttatccatgctg
cacatacataacatatcaagatcagaacacacatatacacacacaaatacaatcaagtcaacaactcc
aaaaagtccagatctacatatatacatacgtaaataacaaaatcatgtaaataatcacaatcatgtaa
tccagatctatgcacatatatatatacacaattaataaaaaaaatgatataacagatctatatctatg
tatgtaacaacacaatcagatgagagaagtgatgttttcagatctgtatacatacaaacacaaacaga
tgaacaattgatacgtagatccatatgtatacgtacaattagctacacgattaaatgaaaaaaatcaa
cgatttcggattggtacacacaaacgcaacaatatgaagaaattcatatctgattagatataaacata
accacgtgtagatacacagtcaaatcaacaaatttatagcttctaaacggatgagatgaacaagataa
agatattcacataaggcatacataagataagcagattaacaaactagcaataatacatacctaattaa
aacaaggaataacagagagagagagagagagagagatttaccttgaaaatgaagaggagaagagagga
tttcttaaaattgggggtagagaaagaaagatgatgaattgtgagaaaggagagatagaaggggggt
tgtatatataggctgtagaagattatttttgtgtttgaggcggtgaaggaagaggggatctgactatg
acacgtttgcggttacgtatttcgataggagtctttcaacgcttaacgccgttactctatatgaccgt
ttgggccgtaacggggccgtttgttaacgctgatgttgattcttttctttctttctttcttcctttt
taaagaagcaattgtacaatcgttgctagctgtcaaacggataattcggatacggatatgcctatatt
catatccgtaattttttcaatctacgctggtctactgagtctaatgccaactttgtacaagaaagctgg
gtctagaaaaaaagcaccgactcggtgccactttttcaagttgataacggactagccttattttaact
tgctatttctagctctaaaacgctctgactgaatctggaatCAATcactacttcgactctagctgtat
ataaactcagcttcgttttcttatctaagcgatgtgggacttttgaagattgttttcaacttaaatgg

gcctatataagaaatactattgttctttcccatataaatgggcctgcttctcttctttcagattccca
ggggccttttgaagattatcttcatatcttaagaatgaagatgttttattcaatcaaattcttgaagg
ttcgatgcctaatcattctaatcctgggacaaactatgaaacaagatacaaaaactccgaatggaaag
ttaaaaagaagaaaacgaaagctacggttcaagaaaatgtaagctgataaacaaaaaaaaactgtatg
aacgaagaagaagaaaaaaagaccgtaatgccaactttgtacaagaaagctgggtctagaaaaaaagc
accgactcggtgccacttttcaagttgataacggactagccttattttaacttgctatttctagctc
taaaacccgaccgtgttcttgctcccCAATcactacttcgactctagctgtatataaactcagcttcg
ttttcttatctaagcgatgtgggacttttgaagattgttttcaacttaaatgggcctatataagaaat
actattgttctttcccatataaatgggcctgcttctcttctttcagattcccaggggccttttgaaga
ttatcttcatatcttaagaatgaagatgttttattcaatcaaattcttgaaggttcgatgcctaatca
ttctaatcctgggacaaactatgaaacaagatacaaaaactccgaatggaaagttaaaaagaagaaaa
cgaaagctacggttcaagaaaatgtaagctgataaacaaaaaaaaactgtatgaacgaagaagaagaa
aaaaagagggtaatgccaactttgtacaagaaagctgggtctagaaaaaaagcaccgactcggtgcca
cttttcaagttgataacggactagccttattttaacttgctatttctagctctaaaaccggcccttt
aatttccttatCAATcactacttcgactctagctgtatataaactcagcttcgttttcttatctaagc
gatgtgggacttttgaagattgttttcaacttaaatgggcctatataagaaatactattgttctttcc
catataaatgggcctgcttctcttctttcagattcccaggggccttttgaagattatcttcatatctt
aagaatgaagatgttttattcaatcaaattcttgaaggttcgatgcctaatcattctaatcctgggac
aaactatgaaacaagatacaaaaactccgaatggaaagttaaaaagaagaaaacgaaagctacggttc
aagaaaatgtaagctgataaacaaaaaaaaactgtatgaacgaagaagaagaaaaaaagaagctaatg
ccaactttgtacaagaaagctgggtctagaaaaaaagcaccgactcggtgccacttttcaagttgat
aacggactagccttattttaacttgctatttctagctctaaaactggcatgtctaagggattgcCAAT
cactacttcgactctagctgtatataaactcagcttcgttttcttatctaagcgatgtgggacttttg
aagattgttttcaacttaaatgggcctatataagaaatactattgttctttcccatataaatgggcct
gcttctcttctttcagattcccaggggccttttgaagattatcttcatatcttaagaatgaagatgtt
ttattcaatcaaattcttgaaggttcgatgcctaatcattctaatcctgggacaaactatgaaacaag
atacaaaaactccgaatggaaagttaaaaagaagaaaacgaaagctacggttcaagaaaatgtaagct
gataaacaaaaaaaaactgtatgaacgaagaagaagaaaaaaaggcaatctactgacctaggccttaa
GGGCCAGATCTTGGGCCCGGTACCCGATCAGATTGTCGTTTCCCGCCTTCGGTTTAAACTATCAGTGT
TT**GACAGGATATATTGGCGGGTAAAC**CTAAGAGAAAAGAGCGTTTATTAGAATAATCGGATATTTAAA
AGGGCGTGAAAAGGTTTATCCGTTCGTCCATTTGTATGTGCATGCCAACCACAGGGTTCCCCTCGGGA
GTGCTTGGCATTCCGTGCGATAATGACTTCTGTTCAACCACCCAAACGTCGGAAAGCCTGACGACGGA
GCAGCATTCCAAAAAGATCCCTTGGCTCGTCTGGGTCGGCTAGAAGGTCGAGTGGGCTGCTGTGGCTT
GATCCCTCAACGCGGTCGCGGACGTAGCGCAGCGCCGAAAAATCCTCGATCGCAAATCCGACGCTGTC
GAAAAGCGTGATCTGCTTGTCGCTCTTTCGGCCGACGTCCTGGCCAGTCATCACGCGCCAAAGTTCCG
TCACAGGATGATCTGGCGCGAGTTGCTGGATCTCGCCTTCAATCCGGGTCTGTGGCGGGAACTCCACG
AAAATATCCGAACGCAGCAAGATATCGCGGTGCATCTCGGTCTTGCCTGGGCAGTCGCCGCCGACGCC
GTTGATGTGGACGCCGAAAAGGATCTAGGTGAAGATCCTTTTTGATAATCTCATGACCAAAATCCCTT
AACGTGAGTTTTCGTTCCACTGAGCGTCAGACCCCGTAGAAAAGATCAAAGGATCTTCTTGAGATCCT
TTTTTTCTGCGCGTAATCTGCTGCTTGCAAACAAAAAAACCACCGCTACCAGCGGTGGTTTGTTTGCC
GGATCAAGAGCTACCAACTCTTTTTCCGAAGGTAACTGGCTTCAGCAGAGCGCAGATACCAAATACTG
TTCTTCTAGTGTAGCCGTAGTTAGGCCACCACTTCAAGAACTCTGTAGCACCGCCTACATACCTCGCT
CTGCTAATCCTGTTACCAGTGGCTGCTGCCAGTGGCGATAAGTCGTGTCTTACCGGGTTGGACTCAAG
ACGATAGTTACCGGATAAGGCGCAGCGGTCGGGCTGAACGGGGGGTTCGTGCACACAGCCCAGCTTGG
AGCGAACGACCTACACCGAACTGAGATACCTACAGCGTGAGCTATGAGAAAGCGCCACGCTTCCCGAA
GGGAGAAAGGCGGACAGGTATCCGccCGGCAGGGTCGGAACAGGAGAGCGCACGAGGGAGCTTCCAGG
GGGAAACGCCTGGTATCTTTATAGTCCTGTCGGGTTTCGCCACCTCTGACTTGAGCGTCGATTTTTGT
GATGCTCGTCAGGGGGGCGGAGCCTATGGAAAAACGCCAGCAACGCGGCCTTTTTACGGTTCCTGGCC
TTTTGCTGGCCTTTTGCTCACATGTTCTTTCCTGCGTTATCCCCTGATTCTGTGGATAACCGATTACC

GCCTTTGAGTGAGCTGATACCGCTCGCCGCAGCCGAACGACCGAGCGCAGCGAGTCAGTGAGCGAGGA
AGCGGAAGAGCGCCTGATGCGGTATTTTCTCCTTACGCATCTGTGCGGTATTTCACACCGCATATGGT
GCACTCTCAGTACAATCTGCTCTGATGCCGCATAGTTAAGCCAGTATACACTCCGCTATCGCTACGTG
ACTGGGTCATGGCTGCGCCCCGACACCCGCCAACACCCGCTGACGCGCCCTGACGGGCTTGTCTGCTC
CCGGCATCCGCTTACAGACAAGCTGTGACCGTCTCCGGGAGCTGCATGTGTCAGAGGTTTTCACCGTC
ATCACCGAAACGCGCGAGGCAGGGGTACGTCGAGGTCGATCCAACCCCTCCGCTGCTATAGTGCAGTC
GGCTTCTGACGTTCAGTGCAGCCGTCTTCTGAAAACGACATGTCGCACAAGTCCTAAGTTACGCGACA
GGCTGCCGCCCTGCCCTTTTCCTGGCGTTTTCTTGTCGCGTGTTTTAGTCGCATAAAGTAGAATACTT
GCGACTAGAACCGGAGACATTACGCCATGAACAAGAGCGCCGCCGCTGGCCTGCTGGGCTATGCCCGC
GTCAGCACCGACGACCAGGACTTGACCAACCAACGGGCCGAACTGCACGCGGCCGGCTGCACCAAGCT
GTTTTCCGAGAAGATCACCGGCACCAGGCGCGACCGCCCGGAGCTGGCCAGGATGCTTGACCACCTAC
GCCCTGGCGACGTTGTGACAGTGACCAGGCTAGACCGCCTGGCCCGCAGCACCCGCGACCTACTGGAC
ATTGCCGAGCGCATCCAGGAGGCCGGCGCGGGCCTGCGTAGCCTGGCAGAGCCGTGGGCCGACACCAC
CACGCCGGCCGGCCGCATGGTGTTGACCGTGTTCGCCGGCATTGCCGAGTTCGAGCGTTCCCTAATCA
TCGACCGCACCCGGAGCGGGCGCGAGGCCGCCAAGGCGCGAGGCGTGAAGTTTGGCCCCCGCCCTACC
CTCACCCCGGCACAGATCGCGCACGCCCGCGAGCTGATCGACCAGGAAGGCCGCACCGTGAAAGAGGC
GGCTGCACTGCTTGGCGTGCATCGCTCGACCCTGTACCGCGCACTTGAGCGCAGCGAGGAAGTGACGC
CCACCGAGGCCAGGCGGCGCGGTGCCTTCCGTGAGGACGCATTGACCGAGGCCGACGCCCTGGCGGCC
GCCGAGAATGAACGCCAAGAGGAACAAGCATGAAACCGCACCAGGACGGCCAGGACGAACCGTTTTTC
ATTACCGAAGAGATCGAGGCGGAGATGATCGCGGCCGGGTACGTGTTCGAGCCGCCCGCGCACGTCTC
AACCGTGCGGCTGCATGAAATCCTGGCCGGTTTGTCTGATGCCAAGCTCGCGGCCTGGCCGGCGAGCT
TGGCCGCTGAAGAAACCGAGCGCCGCCGTCTAAAAAGGTGATGTGTATTTGAGTAAAACAGCTTGCGT
CATGCGGTCGCTGCGTATATGATGCGATGAGTAAATAAACAAATACGCAAGGGGAACGCATGAAGGTT
ATCGCTGTACTTAACCAGAAAGGCGGGTCAGGCAAGACGACCATCGCAACCCATCTAGCCCGCGCCCT
GCAACTCGCCGGGGCCGATGTTCTGTTAGTCGATTCCGATCCCCAGGGCAGTGCCCGCGATTGGGCGG
CCGTGCGGGAAGATCAACCGCTAACCGTTGTCGGCATCGACCGCCCGACGATTGACCGCGACGTGAAG
GCCATCGGCCGGCGCGACTTCGTAGTGATCGACGGAGCGCCCCAGGCGGCGGACTTGGCTGTGTCCGC
GATCAAGGCAGCCGACTTCGTGCTGATTCCGGTGCAGCCAAGCCCTTACGACATATGGGCCACCGCCG
ACCTGGTGGAGCTGGTTAAGCAGCGCATTGAGGTCACGGATGGAAGGCTACAAGCGGCCTTTGTCGTG
TCGCGGGCGATCAAAGGCACGCGCATCGGCGGTGAGGTTGCCGAGGCGCTGGCCGGGTACGAGCTGCC
CATTCTTGAGTCCCGTATCACGCAGCGCGTGAGCTACCCAGGCACTGCCGCCGCCGGCACAACCGTTC
TTGAATCAGAACCCGAGGGCGACGCTGCCCGCGAGGTCCAGGCGCTGGCCGCTGAAATTAAATCAAAA
CTCATTTGAGTTAATGAGGTAAAGAGAAAATGAGCAAAAGCACAAACACGCTAAGTGCCGGCCGTCCG
AGCGCACGCAGCAGCAAGGCTGCAACGTTGGCCAGCCTGGCAGACACGCCAGCCATGAAGCGGGTCAA
CTTTCAGTTGCCGGCGGAGGATCACACCAAGCTGAAGATGTACGCGGTACGCCAAGGCAAGACCATTA
CCGAGCTGCTATCTGAATACATCGCGCAGCTACCAGAGTAAATGAGCAAATGAATAAATGAGTAGATG
AATTTTAGCGGCTAAAGGAGGCGGCATGGAAAATCAAGAACAACCAGGCACCGACGCCGTGGAATGCC
CCATGTGTGGAGGAACGGGCGGTTGGCCAGGCGTAAGCGGCTGGGTTGTCTGCCGGCCCTGCAATGGC
ACTGGAACCCCCAAGCCCGAGGAATCGGCGTGAGCGGTCGCAAACCATCCGGCCCGGTACAAATCGGC
GCGGCGCTGGGTGATGACCTGGTGGAGAAGTTGAAGGCGGCGCAGGCCGCCCAGCGGCAACGCATCGA
GGCAGAAGCACGCCCCGGTGAATCGTGGCAAGCGGCCGCTGATCGAATCCGCAAAGAATCCCGGCAAC
CGCCGGCAGCCGGTGCGCCGTCGATTAGGAAGCCGCCCAAGGGCGACGAGCAACCAGATTTTTTCGTT
CCGATGCTCTATGACGTGGGCACCCGCGATAGTCGCAGCATCATGGACGTGGCCGTTTTCCGTCTGTC
GAAGCGTGACCGACGAGCTGGCGAGGTGATCCGCTACGAGCTTCCAGACGGGCACGTAGAGGTTTCCG
CAGGGCCGGCCGGCATGGCGAGTGTGTGGGATTACGACCTGGTACTGATGGCGGTTTCCCATCTAACC
GAATCCATGAACCGATACCGGGAAGGGAAGGGAGACAAGCCCGGCCGCGTGTTCCGTCCACACGTTGC
GGACGTACTCAAGTTCTGCCGGCGAGCCGATGGCGGAAAGCAGAAAGACGACCTGGTAGAAACCTGCA
TTCGGTTAAACACCACGCACGTTGCCATGCAGCGTACGAAGAAGGCCAAGAACGGCCGCCTGGTGACG
GTATCCGAGGGTGAAGCCTTGATTAGCCGCTACAAGATCGTAAAGAGCGAAACCGGGCGGCCGGAGTA

CATCGAGATCGAGCTAGCTGATTGGATGTACCGCGAGATCACAGAAGGCAAGAACCCGGACGTGCTGA
CGGTTCACCCCGATTACTTTTGATCGATCCCGGCATCGGCCGTTTTCTCTACCGCCTGGCACGCCGC
GCCGCAGGCAAGGCAGAAGCCAGATGGTTGTTCAAGACGATCTACGAACGCAGTGGCAGCGCCGGAGA
GTTCAAGAAGTTCTGTTTCACCGTGCGCAAGCTGATCGGGTCAAATGACCTGCCGGAGTACGATTTGA
AGGAGGAGGCGGGGCAGGCTGGCCCGATCCTAGTCATGCGCTACCGCAACCTGATCGAGGGCGAAGCA
TCCGCCGGTTCCTAATGTACGGAGCAGATGCTAGGGCAAATTGCCCTAGCAGGGGAAAAGGTCGAAA
AGGTCTCTTTCCTGTGGATAGCACGTACATTGGGAACCCAAAGCCGTACATTGGGAACCGGAACCCGT
ACATTGGGAACCCAAAGCCGTACATTGGGAACCGGTCACACATGTAAGTGACTGATATAAAAGAGAAA
AAAGGCGATTTTTCCGCCTAAAACTCTTTAAAACTTATTAAAACTCTTAAAACCCGCCTGGCCTGTGC
ATAACTGTCTGGCCAGCGCACAGCCGAAGAGCTGCAAAAAGCGCCTACCCTTCGGTCGCTGCGCTCCC
TACGCCCCGCCGCTTCGCGTCGGCCTATCGCGGCCGCTGGCCGCTCAAAAATGGCTGGCCTACGGCCA
GGCAATCTACCAGGGCGCGGACAAGCCGCGCCGTCGCCACTCGACCGCCGGCGCCCACATCAAGGCAC
CGGTGGGTATGCCTGACGATGCGTGGAGACCGAAACCTTGCGCTCGTTCGCCAGCCAGGACAGAAATG
CCTCGACTTCGCTGCTGCCCAAGGTTGCCGGGTGACGCACACCGTGGAAACGGATGAAGGCACGAACC
CAGTGGACATAAGCCTGTTCGGTTCGTAAGCTGTAATGCAAGTAGCGTATGCGCTCACGCAACTGGTC
CAGAACCTTGACCGAACGCAGCGGTGGTAACGGCGCAGTGGCGGTTTTCATGGCTTGTTATGACTGTT
TTTTTGGGGTACAGTCTATGCCTCGGGCATCCAAGCAGCAAGCGCGTTACGCCGTGGGTCGATGTTTG
ATGTTATGGAGCAGCAACGATGTTACGCAGCAGGGCAGTCGCCCTAAAACAAAGTTAAACATCATGAG
GGAAGCGGTGATCGCCGAAGTATCGACTCAACTATCAGAGGTAGTTGGCGTCATCGAGCGCCATCTCG
AACCGACGTTGCTGGCCGTACATTTGTACGGCTCCGCAGTGGATGGCGGCCTGAAGCCACACAGTGAT
ATTGATTTGCTGGTTACGGTGACCGTAAGGCTTGATGAAACAACGCGGCGAGCTTTGATCAACGACCT
TTTGGAAACTTCGGCTTCCCCTGGAGAGAGCGAGATTCTCCGCGCTGTAGAAGTCACCATTGTTGTGC
ACGACGACATCATTCCGTGGCGTTATCCAGCTAAGCGCGAACTGCAATTTGGAGAATGGCAGCGCAAT
GACATTCTTGCAGGTATCTTCGAGCCAGCCACGATCGACATTGATCTGGCTATCTTGCTGACAAAAGC
AAGAGAACATAGCGTTGCCTTGGTAGGTCCAGCGGCGGAGGAACTCTTTGATCCGGTTCCTGAACAGG
ATCTATTTGAGGCGCTAAATGAAACCTTAACGCTATGGAACTCGCCGCCCGACTGGGCTGGCGATGAG
CGAAATGTAGTGCTTACGTTGTCCCGCATTTGGTACAGCGCAGTAACCGGCAAAATCGCGCCGAAGGA
TGTCGCTGCCGACTGGGCAATGGAGCGCCTGCCGGCCCAGTATCAGCCCGTCATACTTGAAGCTAGAC
AGGCTTATCTTGGACAAGAAGAAGATCGCTTGGCCTCGCGCGCAGATCAGTTGGAAGAATTTGTCCAC
TACGTGAAAGGCGAGATCACCAAGGTAGTCGGCAAATAATGTCTAACAATTCGTTCAAGCCGACGCCG
CTTCGCGGCGCGGCTTAACTCAAGCGTTAGATGCACTAAGCACATAATTGCTCACAGCCAAACTATCA
GGTCAAGTCTGCTTTTATTATTTTTAAGCGTGCATAATAAGCCCTACACAAATTGGGAGATATATCAT
GAAAGGCTGGCTTTTTCTTGTTATCGCAATAGTTGGCGAAGTAATCGCAACATAGCTTGCTTGGTCGT
TCCGCGTGAACGTCGGCTCGATTGTACCTGCGTTCAAATACTTTGCGATCGTGTTGCGCGCCTGCCCG
GTGCGTCGGCTGATCTCACGGATCGACTGCTTCTCTCGCAACGCCATCCGACGGATGATGTTTAAAAG
TCCCATGTGGATCACTCCGTTGCCCCGTCGCTCACCGTGTTGGGGGGAAGGTGCACATGGCTCAGTTC
TCAATGGAAATTATCTGCCTAACCGGCTCAGTTCTGCGTAGAAACCAACATGCAAGCTCCACCGGGTG
CAAAGCGGCAGCGGC**GGCAGGATATATTCAATTGTAAAT**GGCTTCATGTCCGGGAAATCTACATGGAT
CAGCAATGAGTATGATGGTCAATATGGAGAAAAAGAAAGAGTAATTACCAATTTTTTTTCAATTCAAA
AATGTAGATGTCCGCAGCGTTATTATAAAATGAAAGTACATTTTGATAAAACGACAAATTACGATCCG
TCGTATTTATAGGCGAAAGCAATAAACAAATTATTCTAATTCGGAAATCTTTATTTCGA
